# Genome-wide analysis reveals the spatiotemporal expression patterns of SOS3 genes in the maize B73 genome in response to salt stress

**DOI:** 10.1186/s12864-021-08287-6

**Published:** 2022-01-16

**Authors:** Yunying Cao, Tingyu Shan, Hui Fang, Kangtai Sun, Wen Shi, Bei Tang, Junping Wu, Kai Wang, Ping Li, Baohua Wang

**Affiliations:** 1 Ministry of Agricultural Scientific Observing and Experimental Station of Maize in Plain Area of Southern Region, School of Life Sciences, Nantong University, Nantong, 226019 Jiangsu China; 2 Nantong Changjiang Seed Co., Ltd, Nantong, 226368 Jiangsu China

**Keywords:** Genome-wide analysis, SOS3 gene family, *Zea mays*, qRT-PCR, Subcellular localization

## Abstract

**Background:**

Salt damage is an important abiotic stress that affects the growth and yield of maize worldwide. As an important member of the salt overly sensitive (SOS) signal transduction pathway, the SOS3 gene family participates in the transmission of stress signals and plays a vital role in improving the salt tolerance of plants.

**Results:**

In this study, we identified 59 SOS3 genes in the maize B73 genome using bioinformatics methods and genome-wide analyses. SOS3 proteins were divided into 5 different subfamilies according to the phylogenetic relationships. A close relationship between the phylogenetic classification and intron mode was observed, with most SOS3 genes in the same group sharing common motifs and similar exon-intron structures in the corresponding genes. These genes were unequally distributed on five chromosomes of B73. A total of six SOS3 genes were identified as repeated genes, and 12 pairs of genes were proven to be segmentally duplicated genes, indicating that gene duplication may play an important role in the expansion of the SOS3 gene family. The expression analysis of 10 genes that were randomly selected from different subgroups suggested that all 10 genes were significantly differentially expressed within 48 h after salt treatment, of which eight SOS3 genes showed a significant decline while *Zm00001d025938* and *Zm00001d049665* did not. By observing the subcellular localization results, we found that most genes were expressed in chloroplasts while some genes were expressed in the cell membrane and nucleus.

**Conclusions:**

Our study provides valuable information for elucidating the evolutionary relationship and functional characteristics of the SOS3 gene family and lays the foundation for further study of the SOS3 gene family in the maize B73 genome.

**Supplementary Information:**

The online version contains supplementary material available at 10.1186/s12864-021-08287-6.

## Background

Maize is a major source of human food, animal feed and bioenergy, and it is considered one of the most important crops worldwide. With the continuous growth of the world population, the problem of future maize supply is becoming increasingly prominent and has been threatened for several reasons, including the increasing demand for food, changes in climate and diverse environmental stresses [1]. Soil salinity is a major problem that limits land usage and crop production, especially for maize, a glycophyte plant that is hypersensitive to salinity stress. Previous studies have found that salt stress delays the differentiation and growth of plant organs and tissues via three main aspects, namely, ion toxicity, osmotic stress and nutritional imbalance, which further inhibit the growth and development of whole plants [2]. Plant salt tolerance also mainly involves three aspects, namely, cell homeostasis maintenance (including ion and osmosis), detoxification (such as removal of reactive oxygen species) and growth regulation (such as cell division and stretching) [2, 3]. When plants are under salt stress, the salt overly sensitive (SOS) pathway is activated to regulate ion homeostasis and improve the sodium tolerance of plants [3].

Zhu et al. [4] used genetic mutation analysis methods, such as fast neutron bombardment, T-DNA mutation and chemical mutation (such as EMS induction), to screen approximately 250,000 *Arabidopsis* seedlings, and over 40 SOS mutant lines were recovered. Five groups of SOS mutants were obtained by root-bending assays of these mutant plants on NaCl-containing agar medium, combined with positional cloning and allelic detection [5]; thus, five salt-tolerant genes, *SOS1*, *SOS2*, *SOS3*, *SOS4* and *SOS5*, were identified [6]. Subsequently, the SOS signal transduction pathway has been shown to be a specific ion stress pathway after careful study of *Arabidopsis* salt-sensitive mutants [7]. Extensive genetic studies identified the SOS pathway for NaCl homeostasis and salt tolerance a few decades ago in *Arabidopsis*. This pathway mainly consisted of three components: SOS1, SOS2 and SOS3. In the SOS protein family of *Arabidopsis*, SOS2 and SOS3 were located in the cytoplasm and regulated the homeostasis of K^+^ and Na^+^ inside and outside the cell by regulating SOS1 on the plasma membrane, which could increase the salt tolerance of plants.

*SOS3* is an essential gene that allows plants to grow under conditions of low K^+^ and high salt stress, and it was also the first gene to be cloned [4]. The *SOS3* gene encodes a calcium-binding protein with three EF arms, and the N-terminus contains a myristoylated sequence that contains an essential amino acid, methionine (Met) [8]. When glycine (Gly) located near Met mutates, myristoylation will be prevented, the calcium binding capacity of SOS3 will be weakened, and the ability of SOS3 to activate SOS2 kinase activity will be repressed [9]. The calcium-binding properties of SOS3 may play an important role in determining the specificity of calcium signals in plants under Na^+^ stress. Additionally, the main feature of the amino acid sequences encoded by the SOS3 genes is that the myristoylated sequence located in the N-terminus can specifically bind to the C-terminus of the SOS2 protein to activate the phosphorylation of the SOS2 kinase [9–11]. The *SOS1* gene is a salt-tolerance effector gene encoding a plasma membrane Na^+^/H^+^ antiporter, and its activity and expression are controlled by the Ca^2+^-responsive SOS3-SOS2 protein kinase complex [12]. Some studies have suggested that the SOS3-SOS2 kinase complex could directly promote Na^+^/H^+^ exchange activity and then regulate the flow of Na^+^ through the cell membrane [2]. Moreover, *AtHKT1*, encoding a Na^+^-preferential transporter in *Arabidopsis*, principally controls Na^+^ delivery to the roots-shoots [13]. The hypersensitivity of *SOS3* and *hkt* double mutants to Na^+^ was significantly lower than that of *SOS3* single mutants, suggesting that the SOS3-SOS2 complex may negatively regulate Na^+^ delivery. In addition, the activities of H^+^-ATPase, H^+^-PPase, and Na^+^/H^+^ reverse transporters on the vacuolar membrane may also be regulated by the SOS3-SOS2 complex [14].

To date, the salt tolerance mechanism of SOS3 has been mostly studied in *Arabidopsis* [15–18]. Recent studies have also shown that overexpression of the SOS3 gene can improve the salt tolerance of plants [19–21]. Ma et al. [22] identified 9 grape SOS3 genes in the grape genome and proved that these genes have an important regulatory role in salt stress. However, our knowledge of the characteristics and function of SOS3 genes under salt stress in maize is limited. Here, we clarified the characteristics of SOS3 genes in the maize B73 genome and analyzed the physicochemical properties, structural characteristics, and locations and patterns of SOS3 gene expression under salt stress conditions. This research provides valuable information for elucidating the function of the SOS3 genes and improving the salt resistance of maize via molecular plant breeding.

## Results

### Identification of SOS3 gene family members in the maize B73 genome

Ninety-three SOS3 genes were obtained from the maize B73 genome using a putative EF hand-type calcium binding domain as a query by HMMER software. After screening, the SOS3 protein encoded by nonrepresentative transcripts was excluded, and CDD, Pfam, and SMART were used to check the remaining sequences for the presence of a complete SOS3 domain. Finally, a total of 59 protein sequences were identified as SOS3 proteins in the maize B73 genome, and the molecular weight and isoelectric point were calculated (Table S3). The results showed that the amino acid lengths of the 59 genes ranged from 80 to 1045, while the molecular weights of the 59 genes ranged from 8772.7 to 111,637.6 Da, with an average of 25,655.01 Da. A positive correlation between the molecular weight and the length of amino acids was observed. The isoelectric point size of the coding protein for most of the identified genes was below 7, indicating that almost all SOS3 proteins were acidic, which provided important information for subsequent protein isolation, purification, and electrophoresis.

### Phylogenetic analysis of the SOS3 genes in the maize B73 genome

**Fig. 1:**
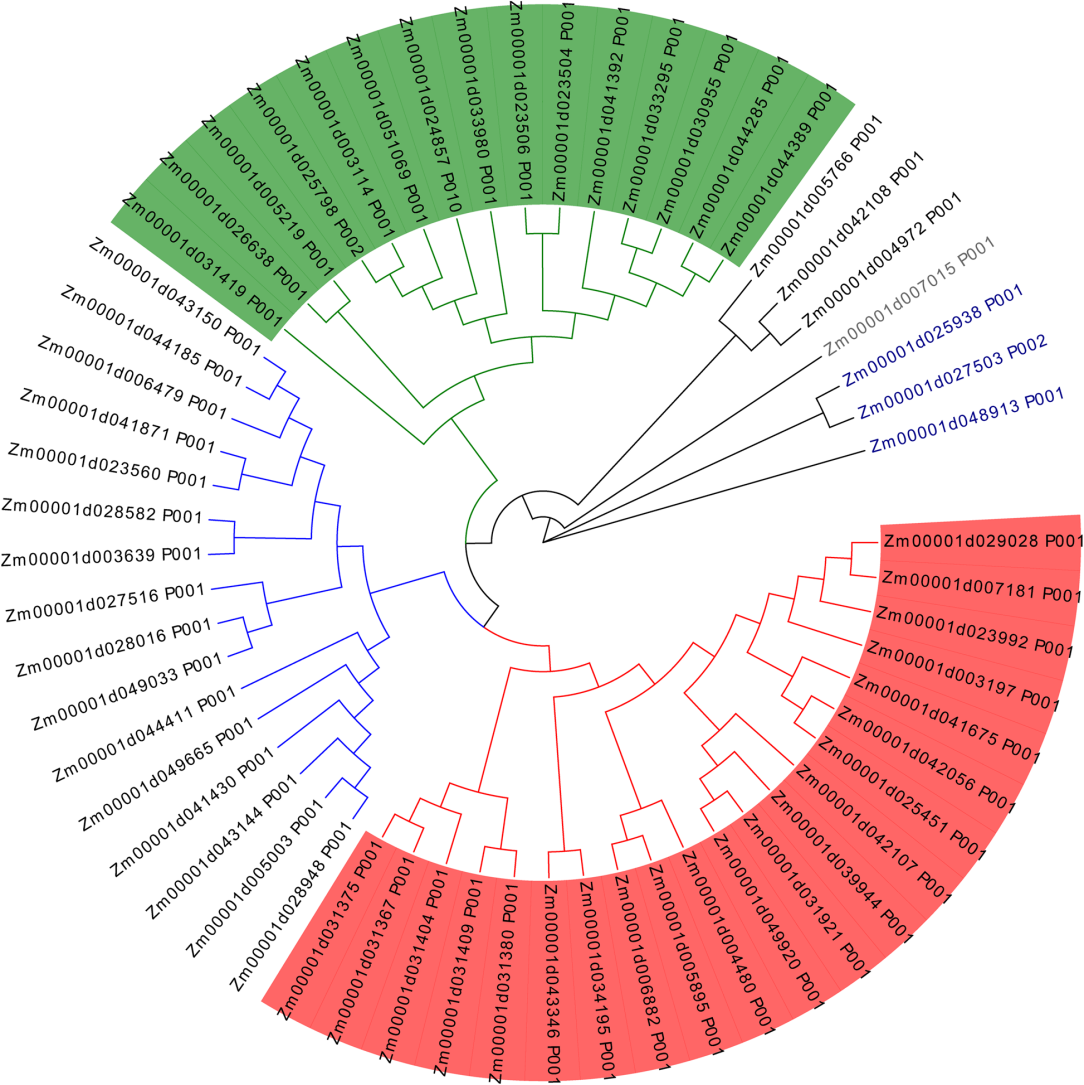
Phylogenetic tree of the SOS3 gene family in maize

To analyze the evolutionary relationship between the members of the SOS3 gene family, sequence alignment of the 59 protein sequences was performed using ClustalW in MEGA7 software; then, a rootless phylogenetic tree was constructed using the maximum likelihood method in MEGA7 software (Fig. 1). As shown in Fig. 1, 59 protein sequences were divided into five different subfamilies. The number of SOS3 genes in the clades ranged from 1 to 21. The group marked in red was the largest subfamily with 21 genes, whereas the group marked in gray was the smallest subfamily with only one gene. To analyze the evolutionary relationships of SOS3 genes in maize, *Arabidopsis*, rice and wheat, an unrooted phylogenetic tree was constructed with full length amino acid sequences (Fig. S2). In total, 59 sequences from maize, 77 sequences from *Arabidopsis*, 69 sequences from rice, and 137 sequences from wheat were assessed in the phylogenetic tree.

### Gene structure, motif and *cis*-acting element analysis of SOS3 genes in the maize B73 genome

**Fig. 2:**
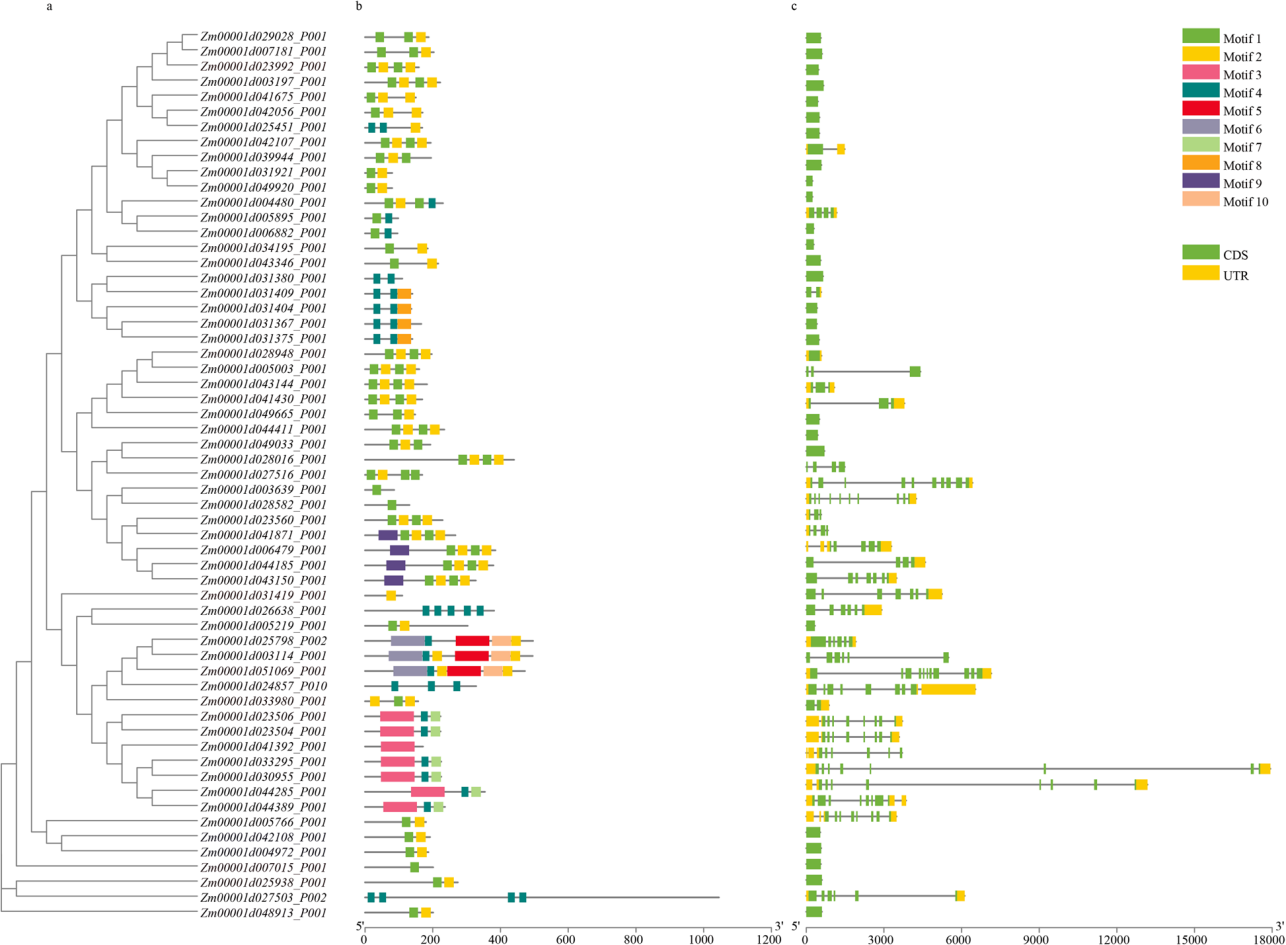
Phylogenetic tree (**a**), consensus motif (**b**), and gene structure (**c**) analysis of the SOS3 gene family in maize B73

The exploration of gene structure is helpful for understanding gene functions and determining the phylogenetic relationships within a gene family. Therefore, the gene structure of the SOS3 genes was identified on the GSDS website. The results showed that the lengths of the SOS3 genes differed greatly, ranging from 243 to 3138. Approximately 45.7% (27/59) of the SOS3 genes were intron-free, while the other genes were disrupted by 1-12 introns, of which three (5.08%) genes contained one intron, and the remaining 29 genes (49.22%) had two or more introns (Fig. 2c). A close relationship was displayed between the phylogenetic classification and gene structure, with most of the SOS3 genes distributed in the same subfamilies possessing similar gene structures. For example, most proteins in the red-labeled group contain only one intron, while most proteins in the blue-labeled group contain multiple introns (Fig. 2a, c). The presence of multiple introns may indicate a specific phylogenetic state. These genes with only one intron or no introns are thought to have lower expression levels in plants. However, the tight genetic structure has the potential to facilitate rapid gene expression in response to endogenous or exogenous stimuli [23]. Subsequently, conserved motifs of the SOS3 proteins were identified through the MEME website, and 10 conserved motifs were identified, ranging from 21 to 100 amino acids in length. The protein sequences of these motifs are presented in Table S4. As shown in Fig. 2b, most of the SOS3 proteins (81.4%) contained either motif 1 or motif 2, while the other SOS3 proteins lacked a complete combination of motifs. Generally, closely related SOS3 proteins in adjacent clades of phylogenetic trees have the same or a similar motif structure.

To obtain information about the *cis*-acting regulatory elements of the SOS3 genes, the putative promoter region sequence of each SOS3 gene was analyzed. A total of 83 putative *cis*-acting regulatory elements were identified by analyzing the 1.5 kb promoter sequences of the SOS3 genes (Fig. S1), and differences existed in the *cis*-acting elements of these genes. Among the *cis*-acting elements identified from the SOS3 promoters, the *Zm00001d005766* promoter contained the most *cis*-acting elements among the 59 genes, with the number of 141. *Zm00001d025798* showed the fewest *cis*-acting elements, with 41 (Fig. S1). The SOS3 genes contained 83 different nonrepetitive *cis*-acting elements that could regulate gene expression. Furthermore, the 83 nonrepetitive *cis*-acting elements were mainly TATA boxes and CAAT boxes, and they were also involved in the light response (ACE, AAAC motif, ATC motif, etc.), gibberellin response (P-box, TATC-box and GARE-motif), abscisic acid response (ABRE), jasmonic acid response (CGTCA-motif and TGACG-motif), salicylic acid response (TCA-element), auxin response (AuxRE and TGA-element), ethylene response (ERE), endosperm expression (GCN4-motif), defense and stress (TC-rich and STRE), and drought induction (LTR) pathways. Among these various *cis*-acting elements, the top 10 with the highest frequency in the SOS3 gene family were TATA-box, CAAT-box, MYB, STRE, G-box, G-Box, CGTCA-motif, TGACG-motif, MYC and as-1.

### Chromosomal mapping of the SOS3 gene family in the maize B73 genome

**Fig. 3:**
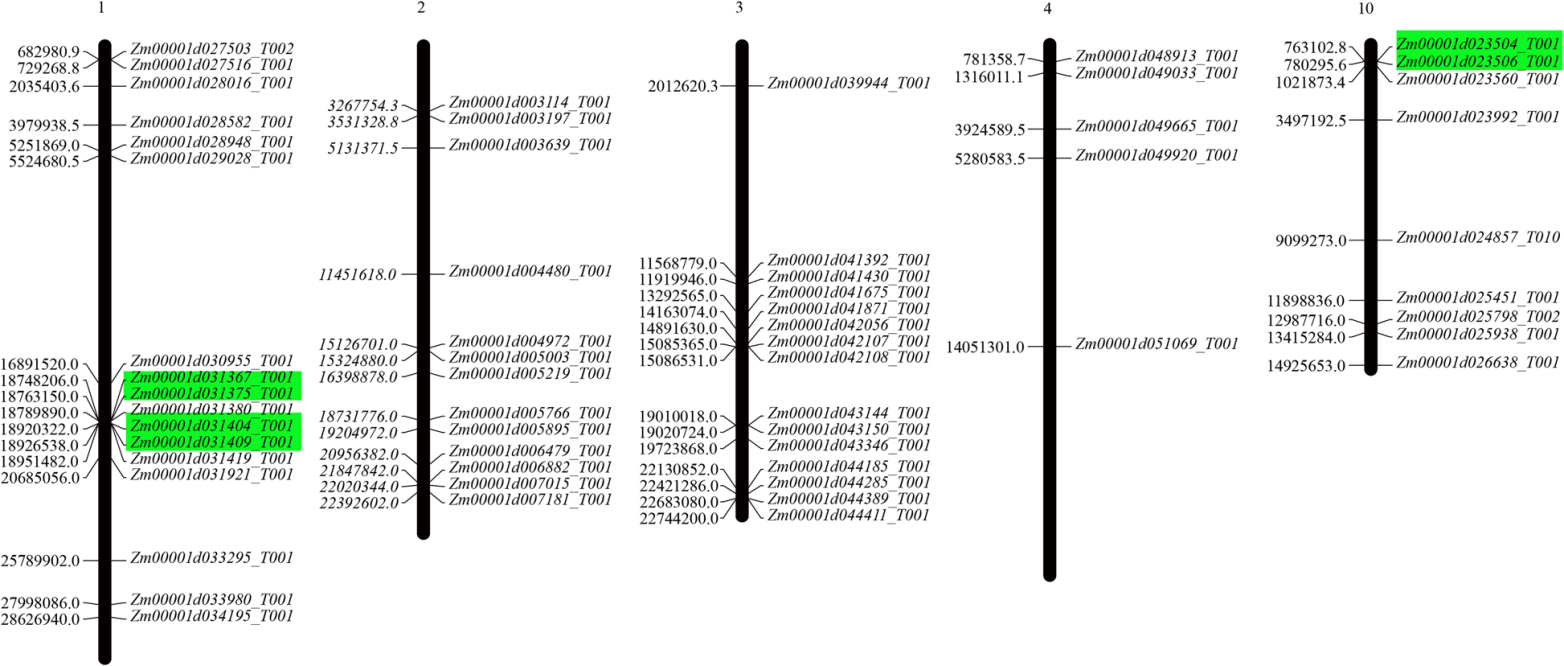
Chromosomal location map of the SOS3 gene family in maize B73. Genes marked in green are tandem repeat genes

To further investigate the genetic divergence and gene duplication events, the SOS3 genes were mapped to maize B73 chromosomes. The results showed that the 59 SOS3 genes were randomly distributed on five chromosomes and displayed an uneven distribution (Fig. 3). The number of genes on each chromosome ranged from 5 to 17; chromosome 1 had the largest number of SOS3 genes at 17, while chromosome 4 had only five SOS3 genes. Additionally, chromosomes 2, 3 and 10 had 13, 15 and 9 SOS3 genes, respectively. Most of these genes were distributed at both ends of chromosomes and were distal to centromeres.

Genome duplication events have always been considered the origin of evolutionary novelties. In maize, because of the bottleneck effect, the majority of these duplicates have been cleared away, and few surviving duplicates have important functions. Gene duplication, tandem repeats and segmental repeats contribute to the generation of gene families during evolution. Therefore, we analyzed the gene duplication events of the SOS3 genes in the maize B73 genome. In total, three pairs (5.1%) of SOS3 genes were identified to be tandem repeat genes with an amino acid identity > 70% and an interval between two genes on the chromosome of less than 100 kb, and these pairs were located on chromosomes 1 and 10 (Fig. 3). In addition, we found that 12 pairs of SOS3 genes were segmented repeats with an amino acid identity of more than 70% (Table S5). These results indicated that segment repeats rather than tandem repeats play a crucial role in contributing to the expansion of the SOS3 gene family.

### Ks analysis of the SOS3 genes in the maize B73 genome

The ratio of the non-synonymous substitution rate to the synonymous mutation rate is an important indicator of the environmental selection pressure experienced by the test coding sequences. If the Ka/Ks of the homologous sequence is less than 1, it means that the pair of homologous sequences has undergone negative purifying selection after gene duplication; if Ka/Ks = 1, it means that the homologous sequences have undergone natural selection after gene duplication; and when Ka/Ks > 1, it means that positive selection occurred [24]. Totally 12 SOS3 gene pairs were analyzed in this research. The results indicated that the Ka/Ks ratios of all the SOS3 orthologs were less than 1, representing negative purifying selection on the SOS3 genes (Table S6).

### Expression profiles of maize SOS3 genes in different tissues

Based on maize transcriptome data of B73 released by the previous studies [25], the expression profiles of 50 SOS3 genes in different tissues were obtained and analyzed for the further investigation of their potential function during the process of growth and development in maize. The results showed that the expression levels of SOS3 genes differed greatly and the expression pattern were mainly divided into three groups. The 13 genes in Group 1 were specifically expressed in some tissues (Fig. S3), of which *Zm00001d005895* was characteristically expressed in roots, shoots and leaves; *Zm00001d005003*, *Zm00001d023992*, *Zm00001d026638* and *Zm00001d007181* were only expressed in pollen and anther, indicating that these genes might be involved in development of pollen and anther. The 22 genes in Group 3 constitutively expressed in most tissues, of which the gene *Zm00001d028948* has a higher expression level in all tissues, implying they were likely to be involved in maize growth and development regulation. The 15 genes in Group 2 are expressed in a relatively low level in all tissues. In addition, most of the genes with the same expression pattern were distributed in the same subfamily.

### Expression patterns of SOS3 genes in response to salt stress

**Fig. 4:**
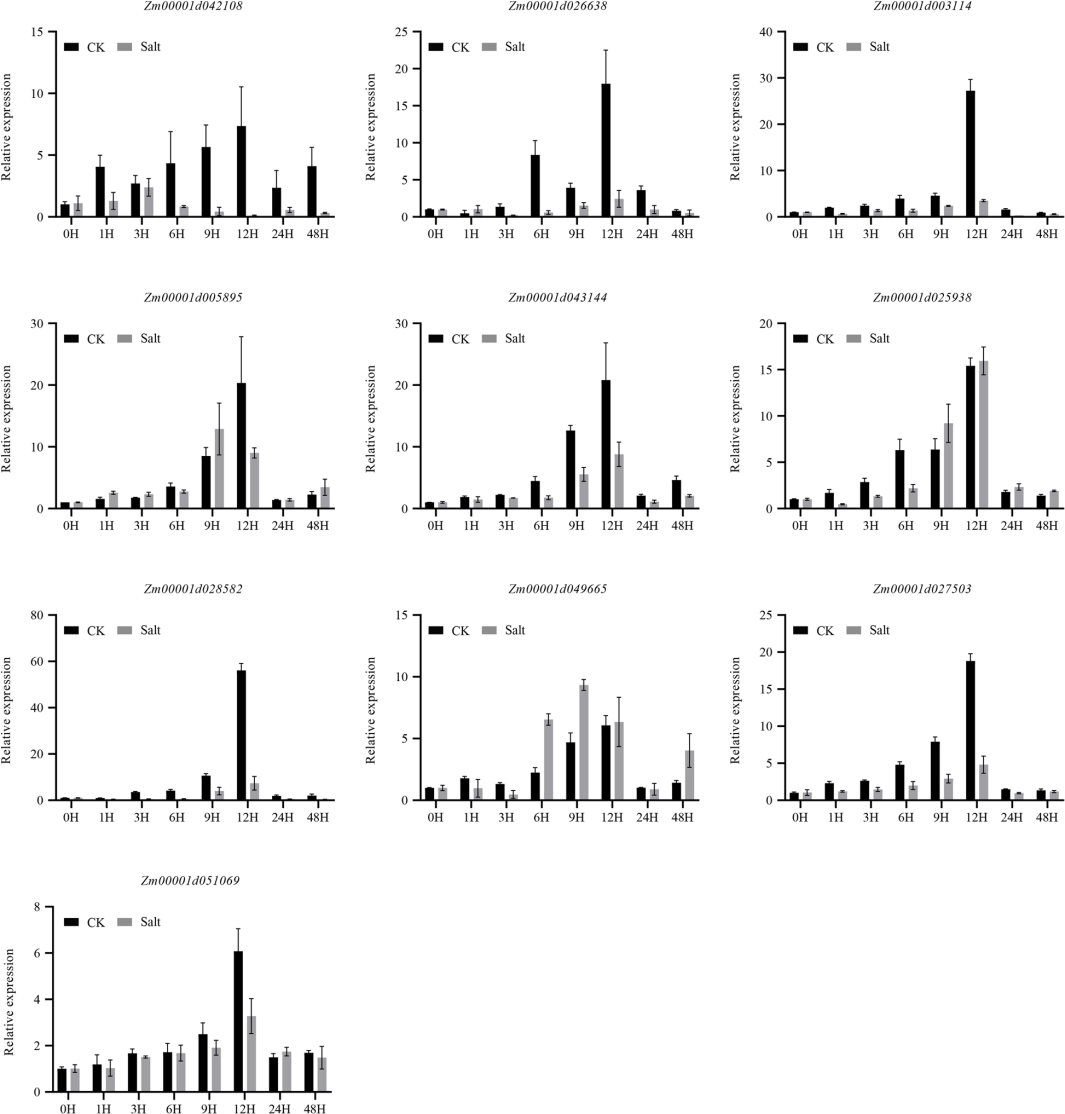
Relative expression levels of 10 candidate genes in maize under salt stress

SOS3 genes have been widely studied and found to play critical roles in response to salt stress in plants. To gain additional insights into the putative roles of SOS3 genes in maize, qRT-PCR was performed to evaluate the transcript abundance of 10 SOS3 genes, that were randomly selected from each subfamily, at different times after salt treatment. Compared with the control, we found that the expression trends of the 10 genes after salt treatment were mostly similar to those of the control (Fig. 4). However, the expression levels of these genes were significantly reduced compared with the control group, indicating that the expression differences in SOS3 genes after salt treatment were caused by salt stress rather than time. Nine of the 10 genes reached the highest expression level under control at 12 h. Eight of the 10 genes displayed the greatest expression decrease after salt treatment at 12 h, including *Zm0001d042108*, *Zm0001d026638*, *Zm00001d003114*, *Zm00001d005895*, *Zm00001d043144*, *Zm00001d028582*, *Zm00001d027503* and *Zm00001d051069*. Of the 8 genes, the expression of *Zm00001d028582* declined after treatment at almost all measured times and barely expressed at several times; the other 6 genes except *Zm00001d051069* also showed expression differences in at least 5 periods. Besides, *Zm00001d025938* displayed a relatively rapid response to salt stress, whose expression decreased at 1 to 6 h after treatment compared to control but increased later. *Zm00001d049665*, unlike any other genes, whose expression decreased at 1-3 h and increased after 6 h under treatment, demonstrated a complex salt stress response pattern.

### Subcellular localization of SOS3 family proteins in maize

**Fig. 5:**
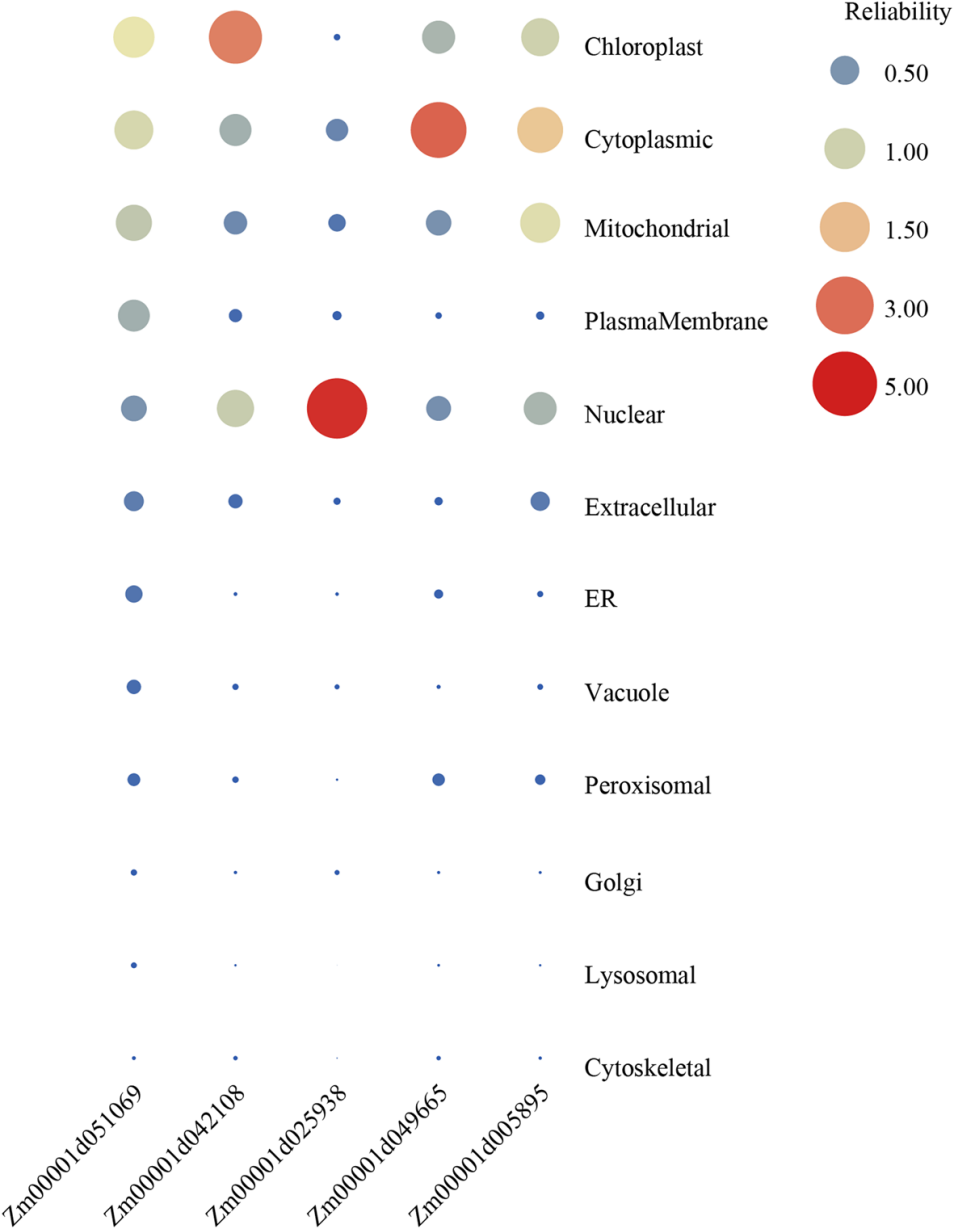
Prediction of subcellular location of candidate genes in tobacco mesophyll cells

**Fig. 6:**
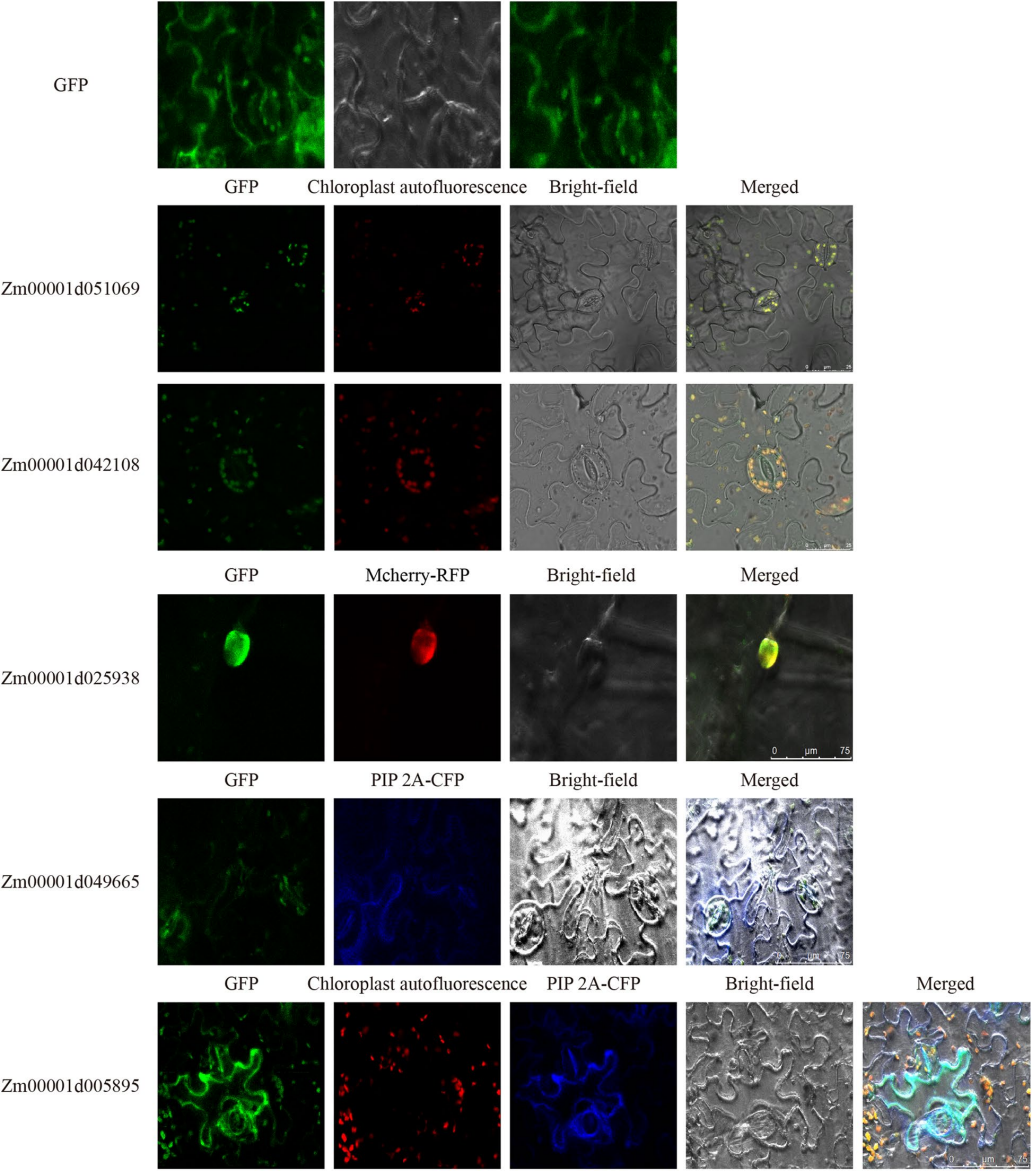
Subcellular localization of candidate genes in tobacco mesophyll cells. The SOS3-GFP fusion protein is predominantly localized to the chloroplast, and some fusion proteins are localized to the cell membrane and nucleus. GFP, green fluorescent protein

The subcellular localizations of proteins are often closely related to their functions. To verify the location of SOS3 proteins, the subcellular localization of 5 SOS3 genes, with one gene randomly selected from each subfamily, was analyzed in tobacco leaves. We first used CELLO to predict the subcellular location of five genes (Fig. 5), and found that *Zm00001d051069* and *Zm00001d042108* were most likely to be localized in chloroplasts, *Zm00001d025938* was predicted to be located on the nuclear, whereas *Zm00001d049665* was possibly to be located in cytoplasmic. In addition, *Zm00001d005895* was likely to be expressed in the cytoplasmic, mitochondrial and nuclear with almost the same reliability according to the predicted results. Subsequently, to identify the precision of bioinformatical subcellular prediction, the fusion proteins of SOS3:GFP were constructed and transiently expressed in tabaco leaves, and then the expression of fusion proteins were observed by confocal microscopy. As shown in Fig. 6, GFP green fluorescence signals were present in the cytoplasm and nucleus, suggesting normal expression of the GFP protein. The GFP signals of the *Zm00001d051069* and *Zm00001d042018* fusion proteins all merged with the chloroplast autofluorescence signal after transient expression in tobacco leaves, thus showing that both genes were localized to chloroplasts. The GFP signal of the *Zm00001d025938* fusion protein merged with the red nucleus-anchored marker protein Mcherry-RFP signal after transient expression in tobacco leaves, indicating the nuclear localization of *Zm00001d025938*. Moreover, the Zm00001d049665 protein was localized to the cell membrane according to the merging of GFP and the cyan membrane-anchored marker PIP 2A-CFP. In addition, the GFP signal of *Zm00001d005895* was observed in chloroplast and cell membrane and coincided with the red chloroplast autofluorescence and cyan PIP 2A-CFP signal in tobacco epidermal cells, indicating that the Zm00001d005895 protein was localized in both the chloroplast and cell membrane. These results are mostly consistent with the predicted results.

## Discussion

Soil salinity is a major abiotic stress factor that seriously affects the growth and development of plants. As one of the most important crops in the world, the development of the maize industry is also facing challenges due to abiotic stress factors, such as soil salinity. It is particularly important to determine the key salt-tolerant genes and breed new salt-tolerant germplasms to improve the salt stress resistance of maize [26].

Among all the salt tolerance mechanisms previously identified in plants, the SOS signal transduction pathway has been most clearly described. The SOS3 gene is an important stress signal sensor in the SOS signal transduction pathway, and it is a special calcium binding protein family [16]. The *SOS3* gene family has been isolated and identified in a few plants, such as *A. thaliana* and grape, and preliminary studies on its function have been carried out [22]; however, research on the SOS3 gene family in maize is still rarely reported.

In this study, 59 protein sequences containing a complete SOS3-specific domain were identified by BLASTP, and their molecular weights and isoelectric points were analyzed. The SOS3 genes in maize encode proteins with different physical and chemical properties and are significantly different in size, function and regulatory mechanism; however, they all have a stable SOS3 domain. Based on the analysis of the phylogenetic tree, we found that the SOS3 proteins were divided into 5 different subfamilies. The number and structure of genes in each subfamily were different. Furthermore, we found that phylogenetic classification was closely related to the gene structure and that most of the SOS3 proteins in the same subfamilies shared common motifs and similar gene structures, which showed consistent arrangements in exons, introns and different motifs.

The analysis of chromosome localization suggested that SOS3 genes were randomly distributed over 5 chromosomes in maize. Gene amplification is a very important driving force in genome evolution and can lead to the emergence of new functional genes and the differentiation of new species, thereby rendering plants more adaptable to harsh environments during evolution [27]. Repetitive sequences are the same or symmetrical DNA sequence fragments that frequently appearing in the plant genome. A single identical fragment is called a repeating unit, and these units account for 10 to 85% of the entire plant genome [28]. Segmented duplicates form due to the process of polyploidization, which preserves many repeated chromosomal fragments in the genome, while tandem duplications are formed by the unequal exchange of nonhomologous sites. Three SOS3 genes were identified to have one duplicate gene, and 12 pairs of genes were proven to be segmented duplicate genes. These duplicate events indicated that segmented repeats might play a more important role than gene duplication in the extension of the SOS3 family.

Identification of the upstream *cis*-acting elements for each gene provides important information on the regulatory mechanisms involved in this process. Among the abiotic stress elements, light response elements were dominant, while among the hormone elements, jasmonic acid was the dominant response element. Previous research reports also indicated that the jasmonic acid signaling pathway was involved in the regulation of the plant salt stress response [29, 30]. Two jasmonic acid related motifs, CGTCA motif and TGACG motif, were unevenly distributed on the promoters of SOS3, suggesting that SOS3 genes may be regulated by salt stress. Additionally, ABRE, a cis-acting element that regulated seed and bud dormancy, could be combined with transcription factors to promote or inhibit the expression of abscisic acid-induced genes, and it has been confirmed in *Arabidopsis* to be related to the stress resistance of plants [31]. Another cis-acting element, named the dehydration-responsive element (DRE), was also found in the promoter regions of many dehydration and cold stress-induced genes. It specifically binds to the DREB transcription factor and plays an important role in inducing the expression of stress-related genes [32]. The *cis*-acting elements differed in SOS3 genes, indicating the complexity of the regulatory mechanism in the SOS3 gene family. Probably, SOS3 family members under evolutionary events such as duplication and insertion/deletion in their sequence in their promoter regions and coding sequence regions have acquired novel function [33, 34].

SOS3 is a Ca^2+^-regulated upstream regulatory protein of the SOS pathway and plays key roles in plant salt stress response pathways [19]. In *Arabidopsis*, the expression of *AtSOS3* was proven to be strongly induced by NaCl treatment [35]. Exploring the expression patterns of SOS3 genes at different times after salt treatment could provide clues to the specific mechanism of these genes in the SOS pathway in maize. We randomly selected 10 genes from different maize SOS3 subfamilies to identify the expression patterns and found that, after salt treatment, the expression of most of the SOS3 genes was downregulated compared with that in the control group with a rapid response to salt stress. Previous studies found that the transcript accumulation of the SOS3 genes increased significantly after 6 h of salt stress treatment in wild-type *Arabidopsis* seedlings [35]. In tomato, after treatment with 200 mM NaCl, the transcription level of *LeENH1*, the enhancer of *SOS3–1* (*ENH1*) that participates in a new salinity stress pathway with SOS2 but without SOS3, gradually increased and reached its highest level in 24 h [36]. These results indicated that the SOS3 genes have a rapid transient expression in response to salt stress, and plant survival under severe stress may require a very immediate cell response [37]. Moreover, SOS3 genes have an important function in the posttranscriptional activation of salt-tolerant effectors [37].

The subcellular localization of the maize SOS3 genes provided useful information for further elucidating the biological functions of the SOS3 genes. A previous study on the subcellular localization of *LeENH1* in tomato found that the green fluorescence of p35S-LeENH1-GFP accumulated in the chloroplast and colocalized with the red autofluorescence of the chloroplast [36]. Recently, five *ThSOS* genes were localized in the plasma membrane, cytoplasm, extracellular space, or chloroplasts [38]. In this study, we found that the SOS proteins were mostly expressed in the chloroplast. The photosynthetic apparatus was easily damaged under salt stress [39]. In chloroplasts, the inhibition of photosynthesis and PSII potentially created a large amount of reactive oxygen species (ROS) under salt stress. When the rate of ROS production was faster than the rate of ROS breakdown, the superfluous ROS derived from chloroplasts transferred into the cytosol and attacked the plasma membrane [40]. Under salt stress, certain proteins of the maize SOS3 gene family were expressed in chloroplasts, and they may have alleviated the inhibition of photosynthesis and PSII photoinhibition to reduce the damage caused by salt stress. In addition, we also observed that some SOS3 proteins were expressed in the cell membrane and nucleus. The SOS3 genes act as Ca^2+^ receptors in plants and are involved in Ca^2+^ signal-mediated stress responses, and myristylation in the N-terminus is important for the recruitment of SOS3 to the plasma membrane [41]. In addition, the difference in SOS3 proteins in subcellular localization implied that these genes may have different functions or may function in different ways when they encounter salt stress.

## Conclusions

In this study, we identified 59 protein sequences containing a complete SOS3-specific domain. The expression analysis of 10 genes that were randomly selected from different subgroups suggested that all 10 genes were significantly differentially expressed within 48 h after salt treatment and most of them were expressed in chloroplasts. This research provides valuable information for elucidating the function of the SOS3 genes and improving the salt resistance of maize via molecular plant breeding.

## Materials and methods

### Plant materials and treatment

The maize B73 inbred line were obtained from National Maize Improvement Center of China. B73, released in 1972, was the product of breeding efforts by Iowa State University agronomy professor emeritus Wilbert Russell [35]. The line and its descendants are now present in half the parentage of nearly all hybrid corn grown around the globe. Plump seeds were picked and treated with 75% ethanol for 1 min and sterile water for 6 h, and then they were placed on moist sterile filter paper and incubated in the dark for germination at 28 °C for approximately 2 days. When the radicles were 1–2 cm in length, the seedlings were transplanted into a small pot with nutrient soil and vermiculite (at a ratio of 3:1) for cultivation in a greenhouse. The temperature and light cycle were set at 24 °C with a 14 h/10 h (light and dark) photoperiod. When the maize seedlings grew to the four-leaf stage, they were divided into two groups: one group was irrigated with 200 ml of water as a blank control, and the other group was irrigated with 200 ml of 250 mmol/L NaCl as the salt treatment. The maize leaves from both the blank control and salt treatment were collected at 0, 1, 3, 6, 9, 12, 24, and 48 h after the salt treatment, immediately frozen in liquid nitrogen and stored in an ultralow temperature refrigerator at − 80 °C for subsequent experiments.

### Identification of SOS3 genes in the maize B73 genome

The database of the maize B73 genome, including the CDSs and protein sequences, was downloaded from the Ensembl Plants website (http://plants.ensembl.org/Zea_mays/Info/Index). The hidden Markov model (HMM) was used to query the SOS3 protein sequences according to the EF-hand domain (PF13499) in Pfam (http://pfam.xfam.org/) throughout the whole B73 genome using the default parameters in HMMER software [42]. After screening, the protein sequences were uploaded to the online software Pfam, the smart database (Simple Modular Architecture Research Tool, SMART, http://smart.embl-heidelberg.de) and the Conserved Domain Database (Conserved domain database, CDD, http://www.NCBI.nlm.NIH.gov/Structure/cdd/wrpsb), and sequence alignment and analysis were carried out to remove unannotated genes and redundant sequences. Subsequently, the sequences with incorrect and nonrepresentative domains were removed. ExPASy Proteomics Server software (http://www.expasy.org) was used to analyze the amino acid sequence of all identified SOS3 genes and calculate the amino acid length and isoelectric point.

### Phylogenetic tree construction and conserved protein motif analysis of SOS3 family genes

The MEGA program (MEGA7) was used to construct a phylogenetic tree using the maximum likelihood method [43]. A bootstrap test (replications) with 1000 iterations was performed. The obtained evolutionary tree was further modified using the Evolview website (http://www.omicsclass.com/article/671). MEME Suite 5.3.3 was applied to carry out motif analysis based on the protein sequences of SOS3 genes, and then the MEME website (http://meme-suite.org/) was used to view the detailed information of the motifs. Finally, TBtools software (https://github.com/CJ-Chen/TBtools) was used to edit and save the obtained motif diagrams.

### Chromosome location and gene duplication analysis of the SOS3 genes

The position information of the SOS3 genes on the chromosome was extracted from the Ensembl Plants website, and BLAST was used to analyze the duplication pattern of each SOS3 gene. Then, the SOS3 genes were mapped to the corresponding chromosome using Mapchart software [44], and chromosome location map was visualized and clarifies using Adobe Illustrator CS6 software.

### Ka/Ks analysis

We calculated the ratio of Ka/Ks to analyze the selection events of genes during the process of evolution. In the course of evolution, the non-synonymous substitutions of amino acids, namely Ka, will lead to the changes in the conformation and function of proteins, resulting in advantages or disadvantages as a result of natural selection; and Ks is the number of SNPs representing synonymous substitutions. The Ka/Ks ratio value of genes were used to carry out species selection pressure analysis. In this study, we used Blast database construction comparison and Calculator tools to calculate the synonymous substitution rate (Ks) and non-synonymous substitution rate (Ka) of the SOS3 gene nucleotides of maize B73 to obtain data, and then proceed to the next step of analysis.

### Gene structure and *cis*-acting element analysis of the SOS3 genes

The gene structure of the SOS3 genes was analyzed using the gene structure display server (GSDS, http://gsds.gao-lab.org/) [45]. To explore the regulation of gene expression, 1.5 kb sequences upstream of the initiation codon of each SOS3 gene were extracted to analyze the *cis*-acting elements of these genes. Plant CARE (*Cis*-Acting Regulatory Element, http://bioinformatics.psb.ugent.be/webtools/plantcare/html/) was used to further analyze the *cis*-acting elements, and the results were mapped using the GSDS online website. The distribution map of the *cis*-acting element in the promoter was drawn via the visualization tool in TBtools.

### Total RNA extraction and qRT-PCR analysis

Total RNA was extracted from B73 leaves at different stages after salt treatment using Takara’s Minibest Universal RNA Extraction Kit (Takara, Kusatsu, Japan). The integrity of the RNA was assessed by 1.0% agarose gel electrophoresis. The Prime Script™ RT Reagent Kit with gDNA Eraser (Takara, Kusatsu, Japan) was used for the synthesis of first strand cDNA. Quantitative real-time polymerase chain reaction (qRT-PCR) assays were carried out in triplicate for each sample using a 2*M5 HIPer Real-time PCR Supermix (SYBRGreen) Kit (Takara, Kusatsu, Japan) with an ABI 7500 real-time PCR system. Relative transcript levels were calculated using the comparative threshold cycle method [46]. The primers for qRT-PCR are listed in Supplementary Table S1.

### Subcellular localization analysis of SOS3 proteins

The CELLO (CELLO: http://cello.life.nctu.edu.tw/) was used to predict the subcellular location of the five selected SOS3 genes, and then perform experiments based on the predicted results. The full-length CDSs of five selected SOS3 genes were amplified by gene-specific primers (Table S2) using KOD One TM PCR Master Mix (TOYOBO, Osaka, Japan) and then subcloned into the commercial pGreen-GFP vector using the ClonExpress® II One Step Cloning Kit (Vazyme, Nanjing, China). The GFP fusion proteins containing the target gene fragment were transferred into Agrobacterium GV3101, and the target gene with Mcherry or PIP 2A was cotransformed into the corresponding tobacco leaves according to the predicted results of the website. Then, the fluorescence images were observed using a Leica TCS SP8 (Mannheim, Germany) confocal microscope imaging system.

## Supplementary Information

**Additional file 1: Figure S1.** Upstream *cis*-acting elements of the SOS3 family genes in maize B73.

**Additional file 2: Figure S2.** Phylogenetic tree of the SOS3 gene family in *Arabidopsis*, rice, wheat and maize.

**Additional file 3: Figure S3.** Expression profiles of maize SOS3 genes in different tissues.

**Additional file 4: Table S1.** Primer sequences for qRT-PCR.

**Additional file 5: Table S2.** Primer sequences for subcellular localization.

**Additional file 6: Table S3.** Information on 59 SOS3 genes in maize B73.

**Additional file 7: Table S4.** List of putative base sequences of SOS3 proteins.

**Additional file 8: Table S5.** SOS3 gene IDs for 12 pairs of segmented repeats.

**Additional file 9: Table S6.** Ka, Ks and Ka/Ks calculation of the SOS3 gene pairs.

## Data Availability

All methods using plant material were carried out in accordance with relevant guidelines and regulations. The data used and/or analyzed during the current study are available from the Ensembl Plants website (http://plants.ensembl.org/Zea_mays/Info/Index). The original contributions presented in the study are included in the article/supplementary material.

## Abbreviations

SOS: Salt overly sensitive
qRT-PCR: Quantitative reverse transcription polymerase chain reaction
ROS: Reactive oxygen species
CDS: Coding DNA sequence
UTR: Untranslated region
