# Supplementary figures and images for: Genome-wide analysis reveals the spatiotemporal expression patterns of SOS3 genes in the maize B73 genome in response to salt stress
Source: BMC Genomics. 2022 Jan 16;23:60. doi: 10.1186/s12864-021-08287-6 (PMC8761280; doi:10.1186/s12864-021-08287-6)

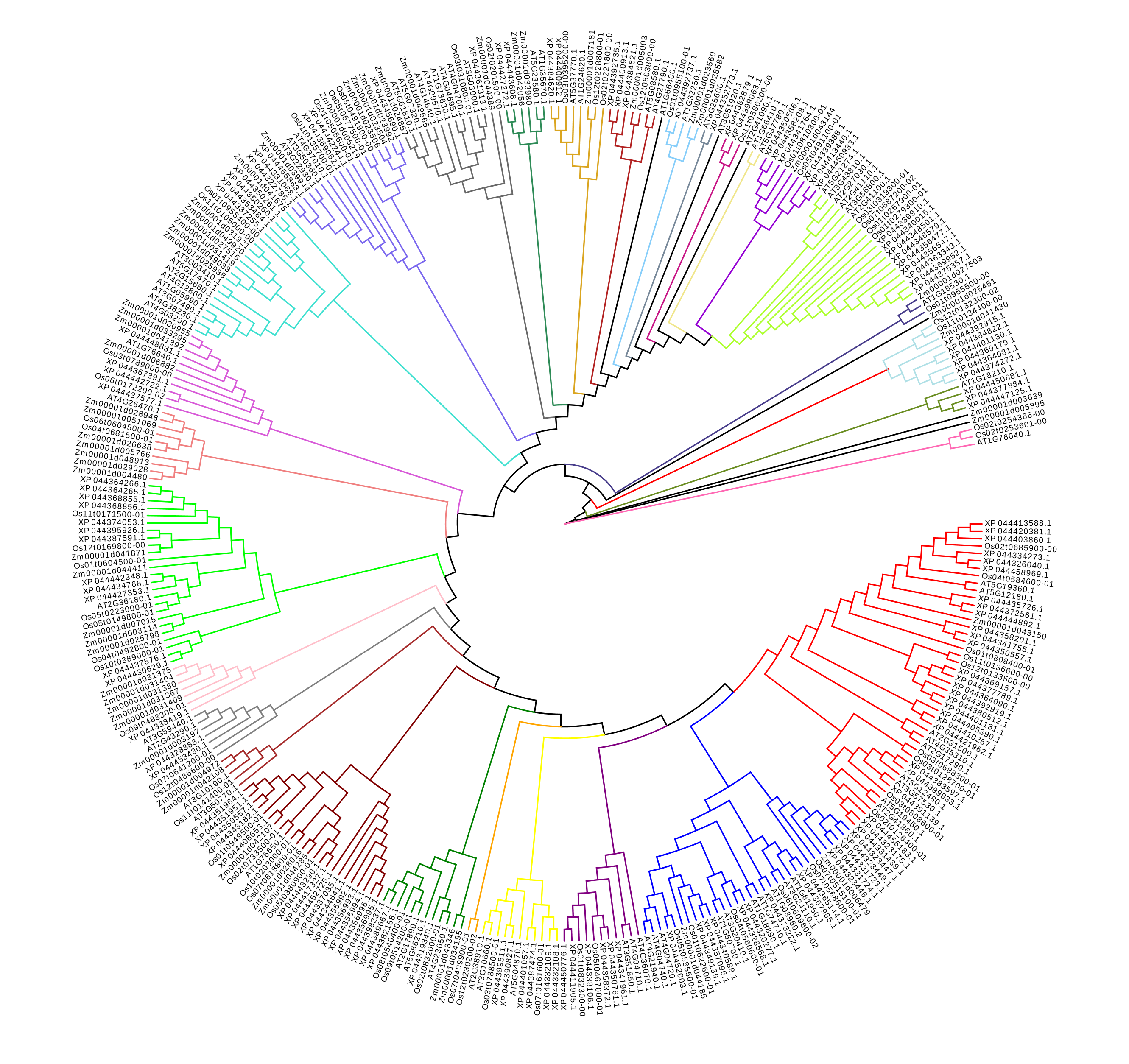

Supplement: Supplementary file 2 — Additional file 2: Figure S2. Phylogenetic tree of the SOS3 gene family in Arabidopsis, rice, wheat and maize. [file 12864_2021_8287_MOESM2_ESM.tiff]

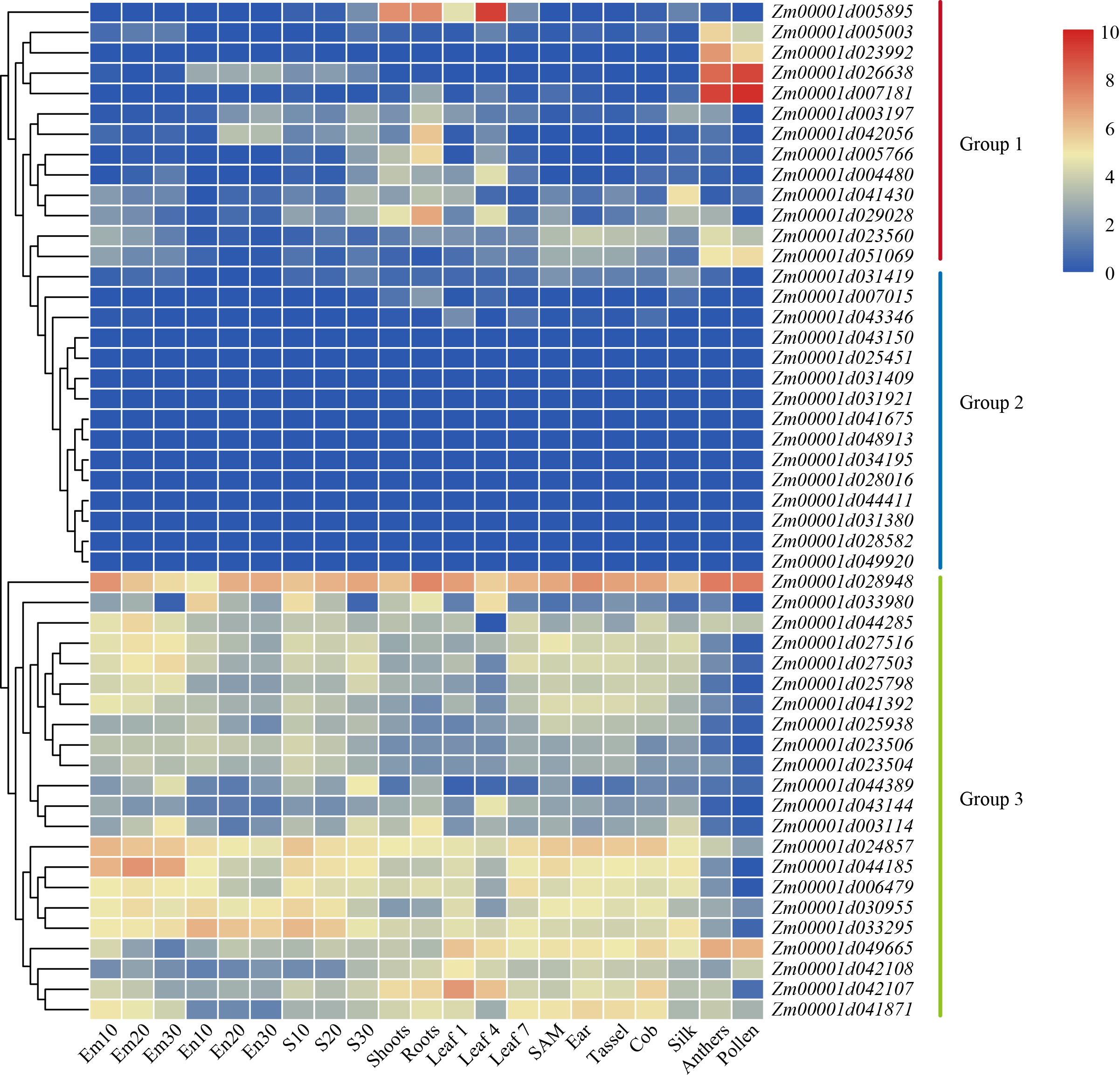

Supplement: Supplementary file 3 — Additional file 3: Figure S3. Expression profiles of maize SOS3 genes in different tissues. [file 12864_2021_8287_MOESM3_ESM.tif]
