# Supplementary material for: Genome-wide analysis reveals the spatiotemporal expression patterns of SOS3 genes in the maize B73 genome in response to salt stress
Source: BMC Genomics. 2022 Jan 16;23:60. doi: 10.1186/s12864-021-08287-6 (PMC8761280; doi:10.1186/s12864-021-08287-6)
Supplement: Supplementary file 4 — Additional file 4: Table S1. Primer sequences for qRT-PCR. [file 12864_2021_8287_MOESM4_ESM.docx]

Table S1 The primers for qRTPCR

| **Primer name** | **Primer sequence** |
| --- | --- |
| ZmActin-F | GCATCCATGAGACCACCTACAAC |
| ZmActin-R | GATGGACCCTCCTATCCAGACAC |
| Zm00001d042108-F | CTGTCCCTGCCGTCCCCGAAGC |
| Zm00001d042108-R | GCGAAGCCCCTGCCGTCCCTGT |
| Zm00001d003114-F | GCACTGATGCGGTCCTTTAAC |
| Zm00001d003114-R | CATTCCCATCACTCCCAAAGA |
| Zm00001d005895-F | GTGGACGAGTTCAAGGAGTGG |
| Zm00001d005895-R | TAACTGATGCCCTGCCTGCT |
| Zm00001d043144-F | ATCGCCGAGGTGGACAGCA |
| Zm00001d043144-R | TGACACGCACGCAGCACCAC |
| Zm00001d025938-F | ACAAGGCACGAATAGGAA |
| Zm00001d025938-R | CCCTTAGTAGGCGTGTTC |
| Zm00001d026638-F | CCTGAAGACAGCAGCCAAGA |
| Zm00001d026638-R | GATGTGAGATTGAGCGACGAA |
| Zm00001d028582-F | GAATGCTGAATCCTCGCTCTG |
| Zm00001d028582-R | ATGCGAGATAAGACAGTTGG |
| Zm00001d049665-F | GCAAGGACCTGACAGAGGAGCA |
| Zm00001d049665-R | TGAAGTCGTCGTAGCGGATGG |
| Zm00001d027503-F | CCAGCCCTTCCTGACTCTCTT |
| Zm00001d027503-R | GCCTCTTTGTGGTAATCCTCCTT |
| Zm00001d051069-F | GTACTTCGCATCCGTCCACA |
| Zm00001d051069-R | AGATGAGGGGAAAACAGGAACA |
