# Supplementary material for: Genome-wide analysis reveals the spatiotemporal expression patterns of SOS3 genes in the maize B73 genome in response to salt stress
Source: BMC Genomics. 2022 Jan 16;23:60. doi: 10.1186/s12864-021-08287-6 (PMC8761280; doi:10.1186/s12864-021-08287-6)
Supplement: Supplementary file 5 — Additional file 5: Table S2. Primer sequences for subcellular localization. [file 12864_2021_8287_MOESM5_ESM.docx]

| **Primer name** | **Primer sequence** |
| --- | --- |
| Zm51069-F | CCTGTTGTTTGGTGTTACTTAAGCTTATGGCTGCCGCGTTAACTCG |
| Zm51069-R | TCCTCGCCCTTGCTCACCATGGATCCCTACTGGGACAGCATCTGTTGAAGAG |
| Zm42108-F | CCTGTTGTTTGGTGTTACTTAAGCTTATGAAGCTGTCCATCCAGTCG |
| Zm42108-R | TCCTCGCCCTTGCTCACCATGGATCCCTAGAAGGCCCGTGCGTC |
| Zm25938-F | CCTGTTGTTTGGTGTTACTTAAGCTTATGGCGGCGGGAAGAGAAAC |
| Zm25938-R | TCCTCGCCCTTGCTCACCATGGATCCTCACTTCTCATGCTCCTGCAACATG |
| Zm49665-F | CCTGTTGTTTGGTGTTACTTAAGCTTATGGGCGGCAAGGACCTGACA |
| Zm49665-R | TCCTCGCCCTTGCTCACCATGGATCCTCATTTGGCGACGATGCGGC |
| Zm05895-F | CCTGTTGTTTGGTGTTACTTAAGCTTATGGCGATCAAGAACATGACGG |
| Zm05895-R | TCCTCGCCCTTGCTCACCATGGATCCTCAGTAGGCGACGATCTTGAGGC |

Table S2 The primers for Subcellular localization
