# Supplementary material for: Genome-wide analysis reveals the spatiotemporal expression patterns of SOS3 genes in the maize B73 genome in response to salt stress
Source: BMC Genomics. 2022 Jan 16;23:60. doi: 10.1186/s12864-021-08287-6 (PMC8761280; doi:10.1186/s12864-021-08287-6)
Supplement: Supplementary file 6 — Additional file 6: Table S3. Information on 59 SOS3 genes in maize B73. [file 12864_2021_8287_MOESM6_ESM.docx]

Table S3 Information of 59 SOS3 genes in maize B73

| Gene ID | AA | MW(Da) | pI |
| --- | --- | --- | --- |
| Zm00001d033295_T001 | 225 | 25819.3 | 4.55 |
| Zm00001d044285_T001 | 354 | 39951 | 5.05 |
| Zm00001d044185_T001 | 379 | 42980.1 | 5.37 |
| Zm00001d031380_T001 | 110 | 12155.4 | 4.48 |
| Zm00001d004480_T001 | 230 | 26808.9 | 5.44 |
| Zm00001d043150_T001 | 327 | 35304.1 | 6.53 |
| Zm00001d029028_T001 | 188 | 20198.5 | 4.24 |
| Zm00001d034195_T001 | 185 | 20083.1 | 7.52 |
| Zm00001d026638_T001 | 381 | 41980.3 | 4.7 |
| Zm00001d025798_T002 | 496 | 55207.7 | 7.57 |
| Zm00001d042056_T001 | 170 | 17689.3 | 4.29 |
| Zm00001d039944_T001 | 195 | 20493.2 | 4.23 |
| Zm00001d003639_T001 | 86 | 9858.4 | 5.13 |
| Zm00001d051069_T001 | 472 | 52573 | 6.59 |
| Zm00001d049665_T001 | 148 | 16638.7 | 4.75 |
| Zm00001d025451_T001 | 169 | 17671.6 | 4.13 |
| Zm00001d023560_T001 | 229 | 25361.5 | 4.49 |
| Zm00001d031921_T001 | 80 | 8846.8 | 4.02 |
| Zm00001d031404_T001 | 137 | 15000.8 | 4.3 |
| Zm00001d028582_T001 | 131 | 15055.3 | 6.94 |
| Zm00001d049920_T001 | 80 | 8772.7 | 4.07 |
| Zm00001d041430_T001 | 169 | 18313 | 3.95 |
| Zm00001d025938_T001 | 274 | 30246.1 | 4.89 |
| Zm00001d005766_T001 | 180 | 18755.5 | 4 |
| Zm00001d031367_T001 | 166 | 18344.6 | 5.32 |
| Zm00001d027503_T002 | 1045 | 111637.6 | 5.52 |
| Zm00001d005895_T001 | 98 | 11253.4 | 4.88 |
| Zm00001d023506_T001 | 223 | 25707.1 | 4.62 |
| Zm00001d031419_T001 | 110 | 12532.5 | 4.08 |
| Zm00001d042108_T001 | 192 | 19810.9 | 4.78 |
| Zm00001d028948_T001 | 197 | 21989.7 | 4.38 |
| Zm00001d044411_T001 | 234 | 24520.2 | 4.49 |
| Zm00001d044389_T001 | 236 | 26992.4 | 4.58 |
| Zm00001d049033_T001 | 193 | 22059.8 | 4.6 |
| Zm00001d007181_T001 | 203 | 21438.6 | 4.1 |
| Zm00001d041871_T001 | 267 | 29061.2 | 4.6 |
| Zm00001d007015_T001 | 201 | 20457.1 | 6.51 |
| Zm00001d030955_T001 | 225 | 25786.1 | 4.55 |
| Zm00001d023992_T001 | 159 | 17642.4 | 4.2 |
| Zm00001d041675_T001 | 150 | 16646.6 | 3.97 |
| Zm00001d023504_T001 | 223 | 25661 | 4.56 |
| Zm00001d031409_T001 | 140 | 15286.2 | 4.31 |
| Zm00001d031375_T001 | 140 | 15311.2 | 4.33 |
| Zm00001d027516_T001 | 169 | 19279.9 | 5.24 |
| Zm00001d042107_T001 | 194 | 20443.6 | 4.54 |
| Zm00001d005219_T001 | 303 | 34188.7 | 4.66 |
| Zm00001d048913_T001 | 201 | 22153.6 | 4.01 |
| Zm00001d004972_T001 | 187 | 19312.1 | 4.36 |
| Zm00001d006479_T001 | 385 | 43040.3 | 4.75 |
| Zm00001d028016_T001 | 440 | 49983.3 | 5.28 |
| Zm00001d024857_T010 | 328 | 38044.8 | 4.49 |
| Zm00001d043144_T001 | 183 | 20462.8 | 4.56 |
| Zm00001d043346_T001 | 216 | 22905 | 4.78 |
| Zm00001d033980_T001 | 157 | 17574.8 | 4.1 |
| Zm00001d005003_T001 | 160 | 18106.6 | 3.92 |
| Zm00001d041392_T001 | 171 | 19453.2 | 4.44 |
| Zm00001d006882_T001 | 96 | 11130.3 | 5.94 |
| Zm00001d003197_T001 | 222 | 24001.3 | 4.18 |
| Zm00001d003114_T001 | 495 | 55662.7 | 8.08 |
