# Supplementary material for: Genome-wide analysis reveals the spatiotemporal expression patterns of SOS3 genes in the maize B73 genome in response to salt stress
Source: BMC Genomics. 2022 Jan 16;23:60. doi: 10.1186/s12864-021-08287-6 (PMC8761280; doi:10.1186/s12864-021-08287-6)
Supplement: Supplementary file 7 — Additional file 7: Table S4. List of putative base sequences of SOS3 proteins. [file 12864_2021_8287_MOESM7_ESM.docx]

Table S4 List of putative base sequences of SOS3 protein

| Motif | Width | Best possible match |
| --- | --- | --- |
| 1 | 26 | ELREAFRVFDKDGDGFITADELRAVL |
| 2 | 29 | SDAEVQEMIREVDADGDGTIDYEEFLAMM |
| 3 | 100 | ALYELFKKISSAVIDDGLINKEEFQLALFKTNKKESLFADRVFDLFDTKHNGILGFEEFARALSVFHPNAPJDEKIDFAFKLYDLKQQGFIERQEVKQMV |
| 4 | 21 | DGLFAEADADGDGKLDRAEFR |
| 5 | 100 | LMRSFNRQGSTHKDGLRIGLKVGQPVENGGVVEYFFGSDGNEPLHFDKFSNFKELHDEIIRLEFSHYDVKSSKTIPAKDFALSMVASADMNHINKLLDR |
| 6 | 100 | ISVACAGATEAREEKGWFLFADSFRRRVFFNYEKRIRLLSPPEKIFEYFASVRNPEGEVYMLPADLMRAVVPVFPPSESTIVREGRLRGERSPGELHCAP |
| 7 | 29 | LLKNMTLPYLKDITTTFPSFVFHSQVDDT |
| 8 | 41 | AEMKEVMLAVANGLGFLPVQMVVEEGSFLKVAVDRELGQLA |
| 9 | 57 | PMEDVRSVYTIGKELGRGQFGVTYLCTHKATGERYACKSIAKRKLTSKEDIEDVRRE |
| 10 | 57 | PDLKDIRITFEEFKAFADLRRRLEPLSMAIFAYGKVNGLLTKEDLKRAAQHVCGVDL |
