# Supplementary material for: Genome-wide analysis reveals the spatiotemporal expression patterns of SOS3 genes in the maize B73 genome in response to salt stress
Source: BMC Genomics. 2022 Jan 16;23:60. doi: 10.1186/s12864-021-08287-6 (PMC8761280; doi:10.1186/s12864-021-08287-6)
Supplement: Supplementary file 8 — Additional file 8: Table S5. SOS3 gene IDs for 12 pairs of segmented repeats. [file 12864_2021_8287_MOESM8_ESM.docx]

Table S5 SOS3 gene IDs for 12 pairs of segmented repeats

| Gene ID | Gene ID |
| --- | --- |
| Zm00001d033295_T001 | Zm00001d030955_T001 |
| Zm00001d033295_T001 | Zm00001d023504_T001 |
| Zm00001d033295_T001 | Zm00001d023506_T001 |
| Zm00001d031921_T001 | Zm00001d049920_T001 |
| Zm00001d031404_T001 | Zm00001d031375_T001 |
| Zm00001d031404_T001 | Zm00001d031367_T001 |
| Zm00001d031367_T001 | Zm00001d031409_T001 |
| Zm00001d023506_T001 | Zm00001d030955_T001 |
| Zm00001d030955_T001 | Zm00001d041392_T001 |
| Zm00001d030955_T001 | Zm00001d023504_T001 |
| Zm00001d031409_T001 | Zm00001d031375_T001 |
| Zm00001d043144_T001 | Zm00001d005003_T001 |
