# Supplementary material for: Genome-wide analysis reveals the spatiotemporal expression patterns of SOS3 genes in the maize B73 genome in response to salt stress
Source: BMC Genomics. 2022 Jan 16;23:60. doi: 10.1186/s12864-021-08287-6 (PMC8761280; doi:10.1186/s12864-021-08287-6)
Supplement: Supplementary file 9 — Additional file 9: Table S6. Ka, Ks and Ka/Ks calculation of the SOS3 gene pairs. [file 12864_2021_8287_MOESM9_ESM.docx]

| Sequence | Ka | Ks | Ka/Ks |
| --- | --- | --- | --- |
| Zm00001d033295&Zm00001d030955 | 0.04367 | 1.17039 | 0.03731 |
| Zm00001d033295&Zm00001d023504 | 0.10208 | 2.28524 | 0.04467 |
| Zm00001d033295&Zm00001d023506 | 0.10094 | 2.17591 | 0.04639 |
| Zm00001d031921&Zm00001d049920 | 0.00692 | 0.737451 | 0.00938 |
| Zm00001d031404&Zm00001d031375 | 0.02127 | 0.224341 | 0.09482 |
| Zm00001d031404&Zm00001d031367 | 0.02454 | 0.307835 | 0.0797 |
| Zm00001d031367&Zm00001d031409 | 0.01344 | 0.270924 | 0.04961 |
| Zm00001d023506&Zm00001d030955 | 0.10616 | 1.91846 | 0.05534 |
| Zm00001d030955&Zm00001d041392 | 0.02704 | 0.207845 | 0.1301 |
| Zm00001d030955&Zm00001d023504 | 0.10646 | 1.94471 | 0.05474 |
| Zm00001d031409&Zm00001d031375 | 0.00587 | 0.117901 | 0.04978 |
| Zm00001d043144&Zm00001d005003 | 0.17579 | 6.0456 | 0.02908 |

Table S6 Ka, Ks and Ka/Ks calculation of the *SOS3* gene pairs
